# Individualized predictions of early isolated distal deep vein thrombosis in patients with acute ischemic stroke: a retrospective study

**DOI:** 10.1186/s12877-021-02088-y

**Published:** 2021-02-25

**Authors:** Hao-Ran Cheng, Gui-Qian Huang, Zi-Qian Wu, Yue-Min Wu, Gang-Qiang Lin, Jia-Ying Song, Yun-Tao Liu, Xiao-Qian Luan, Zheng-Zhong Yuan, Wen-Zong Zhu, Jin-Cai He, Zhen Wang

**Affiliations:** 1 Department of Neurology, the First Affiliated Hospital of Wenzhou Medical University, Wenzhou, 325000 Zhejiang China; 2 Department of Neurology, Wenzhou Hospital of Traditional Chinese Medicine Affiliated to Zhejiang Chinese Medical University, Wenzhou, 325000 Zhejiang China; 3 School of Mental Health, Wenzhou Medical University, Wenzhou, 325000 Zhejiang China; 4 Department of Traditional Chinese Medicine, the First Affiliated Hospital of Wenzhou Medical University, Wenzhou, 325000 Zhejiang China

**Keywords:** Deep vein thrombosis, Nomogram, Prediction, Stroke

## Abstract

**Background:**

Although isolated distal deep vein thrombosis (IDDVT) is a clinical complication for acute ischemic stroke (AIS) patients, very few clinicians value it and few methods can predict early IDDVT. This study aimed to establish and validate an individualized predictive nomogram for the risk of early IDDVT in AIS patients.

**Methods:**

This study enrolled 647 consecutive AIS patients who were randomly divided into a training cohort (*n* = 431) and a validation cohort (*n* = 216). Based on logistic analyses in training cohort, a nomogram was constructed to predict early IDDVT. The nomogram was then validated using area under the receiver operating characteristic curve (AUROC) and calibration plots.

**Results:**

The multivariate logistic regression analysis revealed that age, gender, lower limb paralysis, current pneumonia, atrial fibrillation and malignant tumor were independent risk factors of early IDDVT; these variables were integrated to construct the nomogram. Calibration plots revealed acceptable agreement between the predicted and actual IDDVT probabilities in both the training and validation cohorts. The nomogram had AUROC values of 0.767 (95% CI: 0.742–0.806) and 0.820 (95% CI: 0.762–0.869) in the training and validation cohorts, respectively. Additionally, in the validation cohort, the AUROC of the nomogram was higher than those of the other scores for predicting IDDVT.

**Conclusions:**

The present nomogram provides clinicians with a novel and easy-to-use tool for the prediction of the individualized risk of IDDVT in the early stages of AIS, which would be helpful to initiate imaging examination and interventions timely.

## Introduction

Deep vein thrombosis (DVT) is a common but preventable complication in hospitalized stroke patients [1–3]. Although the incidence of clinically evident DVT after stroke is generally between 2 and 20% [4, 5], this rate has been reported to increase to 75% in immobilized post-stroke patients who do not receive prophylaxis [6]. In the absence of preventive measures, first-time DVT may occur as early as the second day after stroke onset and occurrence peaks from 2 to 7 days post-stroke [5, 7].

In particular, post-stroke DVT predominantly affects the paretic or plegic leg [5] and approximately two-thirds of cases are observed below the knee [8]. Isolated distal deep vein thrombosis (IDDVT) is thrombosis restricted to the infra-popliteal deep veins that account for approximately 23–59% of all DVT cases [9]. The incidence of IDDVT in inpatients range from 12 to 17%, and it can lead to poor prognosis including high rate of death and the recurrence rate of venous thromboembolism (VTE) [10–13]. Meanwhile, a review summarized that about 25–33% of IDDVT patients without treatment extended proximally, which would lead to a high risk of pulmonary embolism (PE) [14]. A prospective study showed that the rate of PE in patients with IDDVT was 8.7% [15]. Besides, long-standing IDDVT is associated with post-thrombotic syndrome (PTS), which presents as venous insufficiency and reduces the patient’s quality of life [16].

The identification of patients with a high risk of early IDDVT is crucial due to its adverse clinical outcomes. Currently, compression ultrasound (CUS) is the most common tool for the diagnosis of IDDVT. It is performed in patients with suspected leg DVT by complete CUS examination of all deep veins [14]. However, the clinical judgment of “suspected” had subjectivity and recent study found the sensitivity and specificity of CUS was not satisfactory for IDDVT [12]. Furthermore, due to the increasing number of asymptomatic post-stroke IDDVT cases [17], CUS may sometimes be relatively too late to detect early IDDVT and perform beneficial interventions, which results in poor clinical outcomes. Currently, due to the lack of objective and reliable clinical signs and symptoms, there are no accurate prediction methods for the early detection of IDDVT to initiate timely CUS monitoring and interventions. Hence, a simple and evidence-based method for assessing a patient’s individual risk of early IDDVT after stroke becomes much needed in the healthcare settings.

Recently, with the advantages of easily accessible format, nomograms have been used to provide accurate risk assessments of specific clinical outcomes for individual patients based on significant factors. Although nomograms appear to be a tool that can assist clinical staff in determining diagnostic and therapeutic strategies for various diseases [18, 19], they have not yet been used for the prediction of IDDVT.

The early detection of patients at a high risk of IDDVT would be helpful for disease management and improving the prognoses of patients. Thus, the present study aimed to establish and validate a predictive nomogram model that could effectively identify early IDDVT risk in acute ischemic stroke (AIS) patients. This would aid clinicians in identifying patients with a higher risk of early IDDVT and enable these patients to receive CUS and appropriate therapies in time.

## Method

### Patient selection

Consecutive AIS patients who presented at the hospital from March 2014 to March 2016 were recruited for this retrospective study using the clinical database of the Department of Neurology at the First Affiliated Hospital of Wenzhou Medical University. The diagnosis of AIS was based on clinical symptoms and confirmed by cranial computerized tomography or magnetic resonance imaging scans within 72 h of admission.

The present study included patients who were hospitalized for more than 7 days, and presented with a neurological deficit at admission (National Institutes of Health Stroke Scale [NIHSS] score ≥ 3). The exclusion criteria were as follows: (1) diagnosis of transient ischemic attacks; (2) discharge prior to the ultrasound or not undergoing an ultrasound; (3) with a history of any central nervous system disease such as Parkinson’s disease, hydrocephalus, or dementia; (4) with a history of VTE; (5) with a history of anticoagulant therapy for other indications; (6) concomitant proximal DVT or symptomatic pulmonary embolism; (7) the presence of severe hepatic or renal diseases; and (8) lack of complete medical records.

**Fig. 1:**
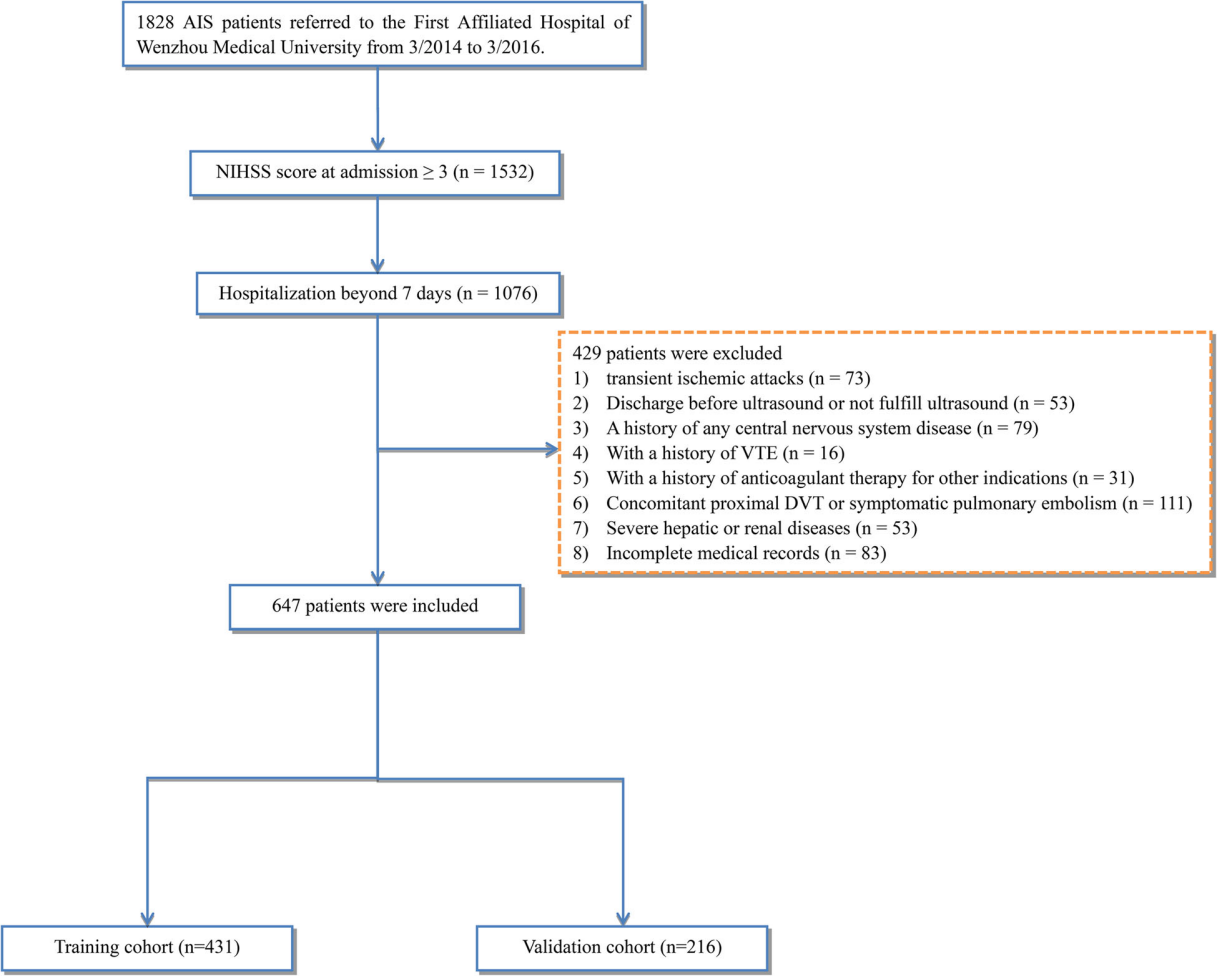
Study flow diagram. AIS, acute ischemic stroke; NIHSS, National Institutes of Health Stroke Scale. DVT, deep vein thrombosis; VTE, venous thromboembolism

Ultimately, 647 AIS patients were eligible for inclusion in this study (Fig. 1). According to the randomized 2:1 assortment procedure, two-thirds of the patients (*n* = 431) were classified into the training cohort, which was used to develop a predictive nomogram model, and the remaining 216 patients were assigned to the validation cohort, which was used to evaluate the performance of the model.

### Data collection

The neurological deficit at admission was based on each patient’s NIHSS score. The lower limb paralysis was defined as lower limbs with NIHSS score of > 2 on item VI [2]. Standard demographic data, including age, gender was collected from the patients’ medical records and clinical parameters, including subtype of stroke, current pneumonia, hypertension, diabetes, current malignant tumor, atrial fibrillation, and current uarthritis, were also assessed. This study only recorded hospital-acquired pneumonia; pneumonia before stroke was not considered. Baseline laboratory parameters, including albumin (ALB) levels, fasting blood glucose levels, serum creatinine concentration (SCr), homocysteine (Hcy) levels, the international normalized ratio (INR), fibrinogen levels, D-Dimer levels, c-reactive protein (CRP) levels, and high-sensitivity C-reactive protein (hs-CRP) levels, were measured within 24 h of admission. Furthermore, the administration of anticoagulant, antiplatelet, lipid-lowering, hormonal and thrombolysis therapies for acute stroke during hospitalization was recorded.

### Whole-leg ultrasonography investigation

A comprehensive real-time B-mode and color Doppler compression ultrasonography examination of both legs was performed routinely at admission and 7 (± 3) days after stroke onset by two experienced vascular physicians who were blind to the baseline health status of each patient. Subsequently, patients with DVT that occurred early in the course of stroke were identified. The diagnosis of DVT was based on the detection of an incompressible segment or an insufficiently compressible lesion in the transverse plane. For the present study, IDDVT was specifically defined as isolated below-knee DVT that occurred below the level of the popliteal vein (anterior tibial veins, posterior tibial veins, peroneal veins, and isolated muscular veins) in the absence of proximal DVT.

### Scoring systems and prognostic models

Several models can be used to predict DVT. The Liu model is a 7-point scoring model that includes age, sex, obesity, active cancer, stroke subtype, and muscle weakness that is highly predictive of ta 14-day risk of DVT in Chinese acute stroke patients [2]. The IMPROVE score, which includes age > 60 years, prior cancer, prior VTE, an intensive care unit or critical care unit stay, lower limb paralysis, immobility, and known thrombophilia state, has a desirable value for predicting the risk of thromboembolism in hospitalized medical patients [20, 21]. Another model used to predict DVT is the Padua Prediction Score, which contained active cancer, previous VTE, reduced mobility, known thrombophilic condition, recent trauma and/or surgery, age ≥ 70 years, heart and/or respiratory failure, acute myocardial infarction or ischemic stroke, acute infection and/or rheumatologic disorder, obesity, and hormonal treatments [22].

### Statistical analysis

All continuous data are presented as the mean ± standard deviation or median with interquartile range, as appropriate, while relative frequencies and proportions are used to express categorical values. Continuous variables were compared using Student’s *t*-test or the Mann–Whitney U test depending on their distributions, while the Chi-square or Fisher’s exact tests were used to compare categorical data. Univariate and multivariate logistic regression analyses were performed to define independent factors that were strongly associated with IDDVT. After adjusting for the main baseline variables that were found to be related to IDDVT in the univariate logistic regression analysis, a multivariate-adjusted binary logistic regression was applied to identify the significant clinical predictors of IDDVT in the training cohort.

Based on the univariate and multivariate logistic models, an IDDVT nomogram was constructed to estimate IDDVT probability; the model was then validated for discrimination and calibration. A validation of the final multivariate model was performed by a bootstrap method with 1000 resamples and the area under the receiver operating characteristic curve (AUROC) was calculated to evaluate the discrimination performance of the IDDVT nomogram in training cohort. Model calibration was assessed by plotting the observed probabilities against the predicted probabilities to determine the predictive accuracy of the nomogram. Additionally, validation was carried out using patients in the validation cohort and the discriminative ability and predictive accuracy performance of the nomogram model were estimated using AUROC and calibration plots. Furthermore, using the AUROC method, the final IDDVT nomogram was compared with other predictive models, including the IMPROVE score, Liu score, and Padua score, to assess its predictive power of early IDDVT.

Two-sided *P* values < 0.05 were considered to indicate statistical significance. All statistical analyses were carried out with SAS statistical software, version 9.2 (SAS Institute Inc., Cary, NC, USA), R 3.4.1 (R Development Core Team, http://www.r-project. org), and MedCalc, version 13.0 (MedCalc Software, Ostend, Belgium).

## Results

### Clinical characteristics of the study cohort

Initially, 647 eligible patients were enrolled in this analysis; of these patients, 142 (21.9%) presented with IDDVT during the first 7 (± 3) days of hospitalization. There were no significant differences (*P* > 0.05) between the training and validation cohorts in terms of basic clinical characteristics or laboratory variables (Table 1). The incidence rates of IDDVT after AIS were similar in the two cohorts: 96 of 431 (22.3%) patients in the training cohort were confirmed with IDDVT and 46 of 216 (21.3%) patients in the validation cohort developed IDDVT.

**Table 1:** Baseline characteristics of AIS patients in training cohort and validation cohort

Variables	Total sample (***n*** = 647)	Training cohort (***n*** = 431)	Validation cohort (***n*** = 216)	***P***-value
IDDVT, n (%)	142 (21.9%)	96 (22.3%)	46 (21.3%)	0.777
**Demographic characteristics**				
Age (years)	68.5 ± 12.1	68.9 ± 12.0	67.7 ± 12.4	0.224
Gender				0.839
male, n (%)	381 (58.9%)	255 (59.2%)	126 (58.3%)	
female, n (%)	266 (41.1%)	176 (40.8%)	90 (41.7%)	
**Subtype of stroke, n (%)**				0.216
Large-artery atherosclerosis	516 (79.8%)	340 (78.9%)	176 (81.5%)	
Cardioembolism	80 (12.4%)	54 (12.5%)	26 (12.0%)	
Small-artery occlusion	6 (0.9%)	2 (0.5%)	4 (1.9%)	
Other or undetermined	25 (3.9%)	20 (4.6%)	5 (2.3%)	
Unknown	20 (3.1%)	15 (3.5%)	5 (2.3%)	
**Clinical parameters, n (%)**				
Lower limb paralysis	248 (38.3%)	169 (39.2%)	79 (36.6%)	0.515
Current pneumonia	132 (20.4%)	84 (19.5%)	48 (22.2%)	0.416
Atrial fibrillation	114 (17.6%)	81 (18.8%)	33 (15.3%)	0.268
Arterial hypertension	504 (77.9%)	327 (75.9%)	177 (81.9%)	0.079
Diabetes mellitus	236 (36.5%)	151 (35.0%)	85 (39.4%)	0.282
Current malignant tumor	34 (5.3%)	21 (4.9%)	13 (6.0%)	0.538
Current uarthritis	27 (4.2%)	17 (3.9%)	10 (4.6%)	0.681
**Laboratory parameters**				
Albumin (g/L)	36.9 ± 4.1	36.9 ± 4.1	37.0 ± 4.0	0.812
Fast blood glucose (mmol/L)	6.0 ± 2.4	5.9 ± 2.3	6.2 ± 2.7	0.255
SCr (μmol/L)	67.0 (55.0–82.0)	67.0 (55.0–82.0)	68.5 (56.0–80.0)	0.814
Hcy (μmol/L)	6.5 ± 8.4	6.1 ± 3.8	7.4 ± 14.0	0.162
INR	1.1 ± 0.3	1.1 ± 0.3	1.1 ± 0.3	0.247
Fibrinogen (g/L)	3.6 ± 1.5	3.6 ± 1.5	3.7 ± 1.4	0.215
D-Dimer (mg/L)	1.3 (0.9–2.6)	1.3 (0.9–2.6)	1.4 (1.0–2.4)	0.675
CRP (mg/L)	8.6 (3.3–23.1)	10.4 (3.4–23.1)	6.0 (3.2–21.4)	0.363
hs-CRP (mg/L)	4.8 (1.1–13.8)	4.2 (1.2–12.8)	5.8 (1.1–21.1)	0.553
**Initial treatment in hospital, n (%)**				
Anticoagulants	200 (30.9%)	138 (32.0%)	62 (28.7%)	0.390
Antiplatelet	546 (84.4%)	358 (83.1%)	188 (87.0%)	0.189
Lipid-lowering agents	627 (96.9%)	417 (96.8%)	210 (97.2%)	0.744
Thrombolysis	76 (11.7%)	56 (13.0%)	20 (9.3%)	0.164
Hormonal treatment	32 (4.9%)	20 (4.6%)	12 (5.6%)	0.613

NOTE. *CRP* c-reactive protein; *Hcy* homocysteine; *hs-CRP* high-sensitivity C-reactive protein; *IDDVT* isolated distal deep venous thrombosis; *SCr* serum creatinine concentration

### Baseline characteristics of patients in the training cohort stratified by IDDVT

The baseline characteristics of the training cohort subgroups with and without IDDVT are presented in Table 2. Compared to patients without IDDVT, patients with IDDVT were more likely to be older (*P* < 0.001; Table 2) and female (*P* = 0.001; Table 2), and showed higher levels of D-Dimer (*P* < 0.001; Table 2), CRP (*P* = 0.008; Table 2) and hs-CRP (*P* = 0.021; Table 2), whereas lower levels of SCr (*P* = 0.016; Table 2). There was also a trend showing that patients with IDDVT had more incidence of lower limb paralysis (59.4% vs. 33.4%; P < 0.001; Table 2). Additionally, more patients in the IDDVT group developed malignant tumor (10.4% vs. 3.3%; *P* = 0.004; Table 2), suffered from pneumonia (36.5% vs. 14.6%; P < 0.001; Table 2), and had atrial fibrillation (34.4% vs. 14.3%; P < 0.001; Table 2).

**Table 2:** Baseline characteristics of AIS patients with IDDVT and Non-IDDVT in training cohort

Variables	Training cohort (n = 431)		
	Non-IDDVT (***n*** = 335)	IDDVT (***n*** = 96)	*P*-value
**Demographic characteristics**			
Age (years)	67.6 ± 12.6	73.4 ± 8.4	< 0.001
Gender			0.001
male, n (%)	212 (63.3%)	43 (44.8%)	
female, n (%)	123 (36.7%)	53 (55.2%)	
**Subtype of stroke, n (%)**			0.248
Large-artery atherosclerosis	271 (80.9%)	69 (71.9%)	
Cardioembolism	36 (10.7%)	18 (18.8%)	
Small-artery occlusion	2 (0.6%)	0	
Other or undetermined	15 (4.5%)	5 (5.2%)	
Unknown	11 (3.3%)	4 (4.2%)	
**Clinical parameters, n (%)**			
Lower limb paralysis	112 (33.4%)	57 (59.4%)	< 0.001
Current pneumonia	49 (14.6%)	35 (36.5%)	< 0.001
Atrial fibrillation	48 (14.3%)	33 (34.4%)	< 0.001
Arterial hypertension	256 (76.4%)	71 (74.0%)	0.620
Diabetes mellitus	115 (34.3%)	36 (37.5%)	0.566
Current malignant tumor	11 (3.3%)	10 (10.4%)	0.004
Current uarthritis	15 (4.5%)	2 (2.1%)	0.288
**Laboratory parameters**			
Albumin (g/L)	37.1 ± 4.2	36.4 ± 4.0	0.170
Fast blood glucose (mmol/L)	5.9 ± 2.2	6.0 ± 2.4	0.556
SCr (μmol/L)	68.0 (57.0–83.0)	63.5 (52.0–78.2)	0.016
Hcy (μmol/L)	6.1 ± 3.8	6.3 ± 3.9	0.697
INR	1.1 ± 0.3	1.1 ± 0.2	0.673
Fibrinogen (g/L)	3.6 ± 1.5	3.6 ± 1.6	0.848
D-Dimer (mg/L)	1.2 (0.8–2.1)	1.8 (1.2–3.7)	< 0.001
CRP (mg/L)	7.3 (3.2–18.6)	14.6 (5.9–31.5)	0.008
hs-CRP (mg/L)	3.5 (1.0–11.8)	8.5 (3.9–32.4)	0.021
**1 n (%)**			
Anticoagulants	101 (30.1%)	37 (38.5%)	0.120
Antiplatelet	281 (83.9%)	77 (80.2%)	0.398
Lipid-lowering agents	324 (96.7%)	93 (96.9%)	0.938
Thrombolysis treatment	12 (3.6%)	8 (8.3%)	0.051

**NOTE**. *CRP* c-reactive protein; *Hcy* homocysteine; *hs-CRP* high-sensitivity C-reactive protein; *IDDVT* isolated distal deep venous thrombosis; *SCr* serum creatinine concentration

### Independent predictors of IDDVT in patients with AIS

A univariate analysis of the training cohort revealed that age, gender, lower limb paralysis, malignant tumor, current pneumonia, and atrial fibrillation were associated with a greater risk of developing early IDDVT after AIS (Table 3). Thus, these factors were entered in the multivariate regression analysis to screen for significant predictors of IDDVT. The final multivariate logistic regression analysis results revealed that these six variables (age, gender, lower limb paralysis, malignant tumor, current pneumonia, and atrial fibrillation) acted as independent predictors of IDDVT among AIS patients (Table 3).

**Table 3:** Univariate and multivariate analysis of the associations between IDDVT and baseline characteristics in training cohort

Variables	Univariate analysis				Multivariate analysis			
	β coefficient	OR	95% CI	*P*-value	β coefficient	OR	95% CI	*P*-value
**Demography parameters**								
Age (years)	0.047	1.048	1.025–1.072	< 0.001	0.028	1.028	1.003–1.054	0.029
Gender (female)	0.753	2.124	1.342–3.364	0.001	0.638	1.893	1.142–3.137	0.013
**Clinical parameters**								
Lower limb paralysis	−1.744	2.910	1.826–4.639	< 0.001	0.987	2.682	1.623–4.434	< 0.001
Current pneumonia	1.209	3.349	2.002–5.601	< 0.001	0.846	2.331	1.321–4.111	0.003
Atrial fibrillation	1.142	3.132	1.861–5.270	< 0.001	0.989	2.689	1.525–4.743	0.001
Arterial hypertension	−0.132	0.876	0.521–1.476	0.620				
Diabetes mellitus	0.138	1.148	0.717–1.838	0.566				
Current malignant tumor	1.231	3.425	1.408–8.330	0.007	1.322	3.750	1.442–9.751	0.007
Current uarthritis	−0.790	0.454	0.102–2.021	0.300				
**Laboratory parameters**								
Albumin (g/L)	−0.038	0.963	0.912–1.016	0.171				
Fast blood glucose (mmol/L)	−0.038	1.030	0.933–1.138	0.555				
SCr (μmol/L)	−0.003	0.997	0.990–1.005	0.473				
Hcy (μmol/L)	0.014	1.014	0.944–1.090	0.696				
INR	−0.184	0.832	0.353–1.960	0.674				
Fibrinogen (g/L)	0.015	1.015	0.875–1.177	0.847				
D-Dimer (mg/L)	0.032	1.033	0.983–1.085	0.200				
CRP (mg/L)	0.004	1.004	0.995–1.012	0.393				
hs-CRP (mg/L)	0.008	1.008	0.997–1.019	0.149				
**Initial treatment in hospital**								
Anticoagulants	0.374	1.453	0.906–2.331	0.121				
Antiplatelet	−0.250	0.779	0.436–1.392	0.399				
Lipid-lowering agents	−1.299	1.052	0.288–3.851	0.938				
Thrombolysis	−1.258	1.064	0.546–2.071	0.856				
Hormonal treatment	−1.300	2.447	0.970–6.172	0.058				

**NOTE**. *CI* confidence interval; *CRP* c-reactive protein; *Hcy* homocysteine; *hs-CRP* high-sensitivity C-reactive protein; *IDDVT* isolated distal deep venous thrombosis; *OR* odds ratio; *SCr* serum creatinine concentration

### Construction of the predictive nomogram for IDDVT in patients with AIS

**Fig. 2:**
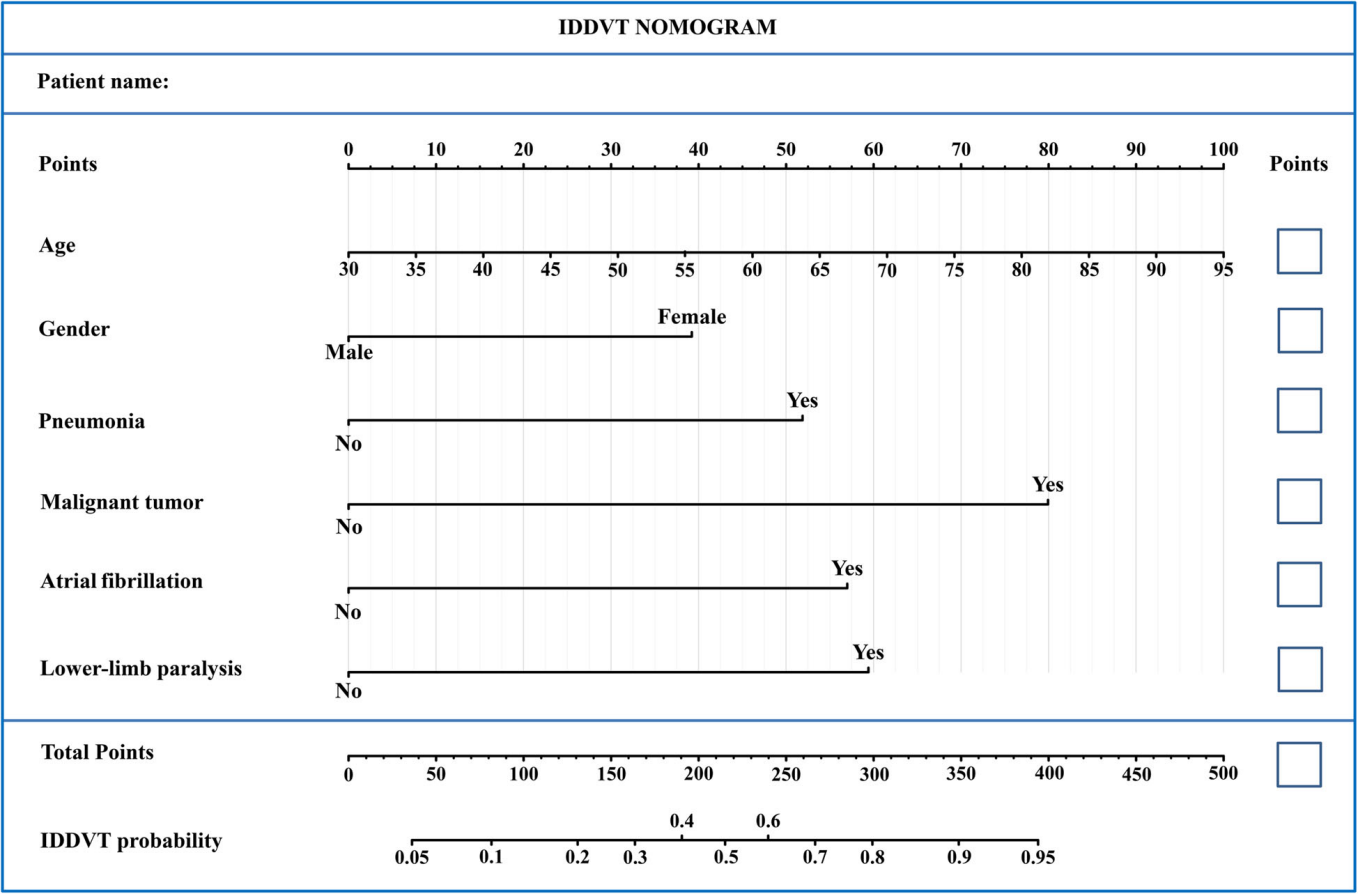
Nomogram model for predicting the risk of early IDDVT in patients with AIS. For all patients, adding up the points identified on the points scale for all six indicators. Then, the sum is located on the “Total Points” axis. Finally, the risk of IDDVT according to the nomogram is the probability of “IDDVT” corresponding to “Total Points”

Based on the six independent predictors of IDDVT identified in the final multivariate logistic regression model, a nomogram for predicting IDDVT in AIS patients was constructed using R software (Fig. 2). Each subtype within these variables corresponded to a matching score on the point scale and, after summing the total scores and positioning them on the total point scale, it was possible to draw a vertical line down to the IDDVT scale to obtain the predicted probability of IDDVT.

### Validation of the predictive nomogram

**Fig. 3:**
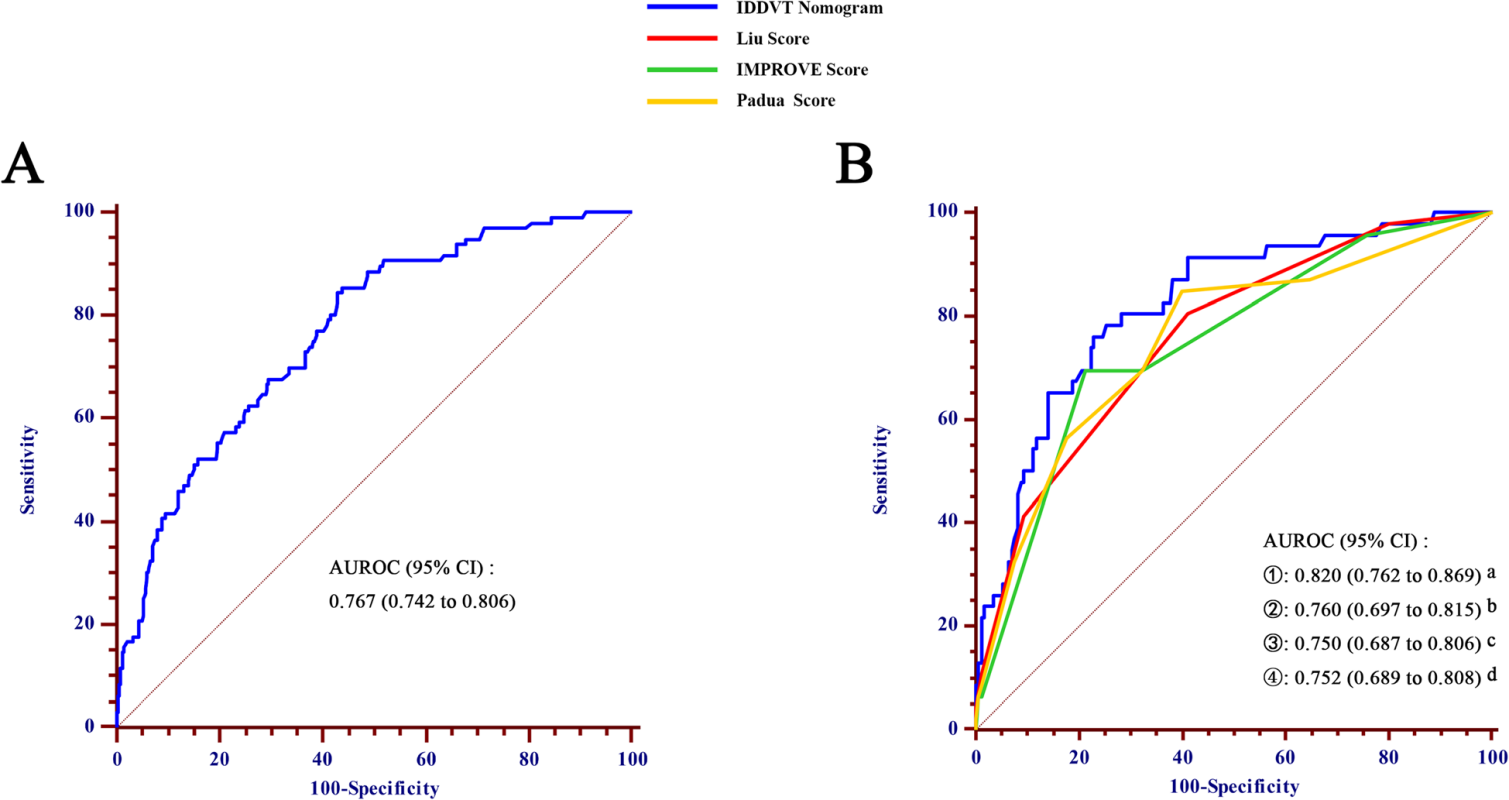
Area under the receiver operating characteristic curve for prediction of IDDVT among different scoring systems in training cohort (panel **a**) and validation cohort (panel **b**). *P* values: a vs. b = 0.024; a vs. c = 0.005; a vs. d = 0.006. AUROC, area under the receiver operating characteristic curve; CI, confidence interval

**Fig. 4:**
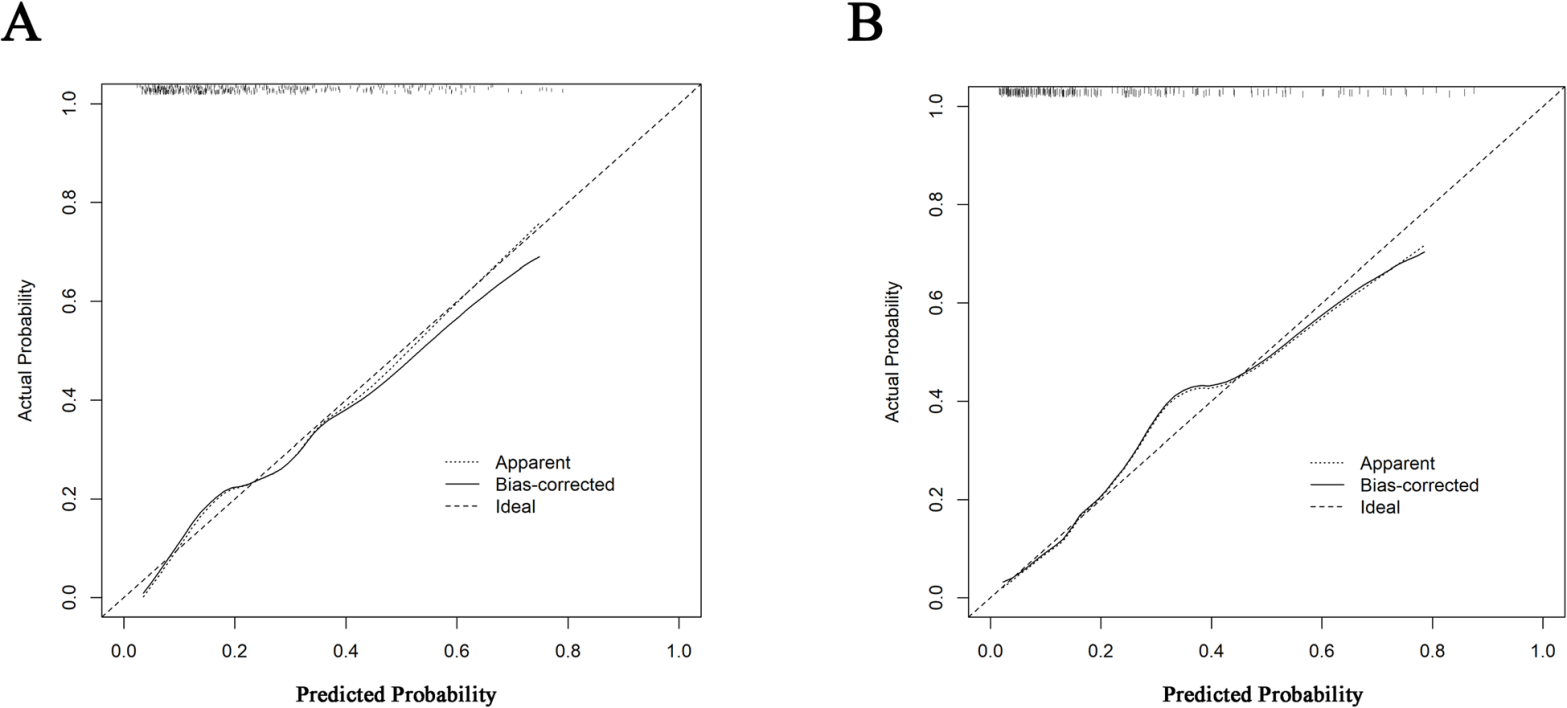
Nomogram calibration plot in training cohort (panel **a**) and validation cohort (panel B). Panel **a**: Mean absolute error = 0.035 (training cohort); panel **b**: Mean absolute error = 0.017 (validation cohort)

Next, a verification of the nomogram was completed in the training cohort. The predictive nomogram exhibited a relatively good discriminative capacity based on an AUROC value of 0.767 (95% CI: 0.742–0.806; Fig. 3a). Additionally, the calibration plot showed that the predicted probability of IDDVT of the nomogram was closely approximated by the actual observations (Fig. 4a). The mean absolute error between the prediction probability and the actual probability in the training cohort was 0.035. Ideally, a completely accurate prediction model would generate a plot on which the observed and predicted probabilities fall along the 45° line [23].

Validation was also assessed in the validation cohort by verifying the comparison between the predicted probability of the nomogram and the actual probability of each patient. The calibration plot revealed good consistency between the predicted and actual probabilities with a mean absolute error of 0.017 between these probabilities (Fig. 4b). The AUROC of the predictive nomogram was 0.820 (95% CI: 0.762–0.869; Fig. 3b).

### Clinical pretest probability assessment

Each patient was also assessed for IDDVT using the Liu score, IMPROVE score, and Padua score to further evaluate the predictive ability of the nomogram for IDDVT. In this result, the AUROC of the nomogram prediction (0.820, 95% CI: 0.762–0.869) was significantly higher than the Liu score prediction (0.760, 95% CI: 0.697–0.815; *P* = 0.024), the IMPROVE prediction (0.750, 95% CI: 0.687–0.806; *P* = 0.005), and the Padua prediction (0.752, 95% CI: 0.689–0.808; *P* = 0.006; Fig. 3b).

## Discussion

In this study, we established and validated a novel and simple nomogram-derived score for predicting the occurrence of early IDDVT in AIS patients within 7 days of stroke onset. And this score was applicable to AIS patients who did not have previous DVT. The IDDVT nomogram prediction model identified several factors that were associated with the patient profile, including age, gender, lower limb paralysis, malignant tumor, pneumonia, and atrial fibrillation. This model exhibited good discrimination in both the training and validation cohorts. The present nomogram model can be easily applied to daily clinical practice and can be helpful for the screening of AIS patients who may be at a high risk of IDDVT, which would be benefit to initiate CUS and interventions timely.

DVT, as a common complication in hospitalized AIS patients, may delay the rehabilitation of stroke, cause postphlebitic leg, varicose ulcers and PE [2]. As is known, IDDVT accounts for approximately 23–59% of all DVT cases [9] and the incidence of IDDVT in inpatients range from 12 to 17%. 25–33% of IDDVTs who without treatment extended proximally [14]. Thus, consistent with DVT, IDDVT could lead to poor prognosis including high rate of death, the recurrence rate of VTE, PE and PTS [10–14, 16]. Meanwhile, proximal DVT (symptomatic) and PE are easily to recognize and diagnosis in time. While IDDVT used to present as asymptomatic and indiscoverable; and the most common tool for the diagnosis of IDDVT is CUS with unsatisfactory sensitivity and specificity [12]. Our predictive nomogram model could effectively help clinicians in identifying patients with a higher risk of early IDDVT and enable these patients to receive CUS and appropriate therapies in time.

Consistent with previous studies, the present findings revealed that gender, age, and a history of atrial fibrillation could contribute to the development of IDDVT. In particular, the present study showed that atrial fibrillation played an important role in the development of DVT. A Northern Norway study found that atrial fibrillation is associated with an increased risk of subsequent VTE [24], which is similar to the findings of a population-based cohort study conducted in China [25]. Because atrial fibrillation can lead to venous stasis, this may lead to a subsequent increase in the risk of VTE through the activation of coagulation factors and platelets [26]. For example, a recent review found that inflammation and various growth factors may play potential roles in promoting a pro-thrombotic state during atrial fibrillation [27]. In terms of gender, many studies have found that female patients are more likely to develop DVT after stroke [2, 28]; this is consistent with our results. Age is also a well-known risk factor of IDDVT that many prediction models have identified as a predictive factor [2, 20–22]. Additionally, some studies have noted that the incidence of VTE is expected to increase with age [29, 30]. The present results also suggest that malignant tumor is commonly related to IDDVT. Cancer is doubtlessly a major risk factor for VTE as it accounts for approximately 20% of VTE cases [31] and several studies found that patients with cancer-related IDDVT are more likely to relapse into VTE [32–34]. Furthermore, a review study concluded that some cancer-associated pathways may be related to the onset of VTE. For example, cancers may increase the risk of VTE by producing neutrophil extracellular traps, inducing monocytes to express tissue factor (TF), and leading to thrombocytosis [35]. Similarly, cancer treatments, such as immunosuppressive or cytotoxic chemotherapy, may have similar effects. A case study of a patient with leukemia who received L-asparaginase therapy described the development of vein thrombosis due to therapy-induced reductions of plasma plasminogen and antithrombin III levels in the patient [36]. Additionally, two placebo-controlled studies showed that lenalidomide and dexamethasone for the treatment of multiple myeloma increase the incidence of venous thrombosis [37].

The present IDDVT prediction model identified lower limb paralysis as a risk factor. A case-control study with a large sample size conducted in the United States found that leg paresis is an independent risk factor of vein thrombosis, with an odds ratio of 6.10 [38]. Moreover, several previous studies have reported that the severity of paralysis is associated with DVT [5, 39]. This may be due to the fact that lower limb paralysis prolongs time spent in a hospital bed and leg immobility time, which may lead to venous stasis [40] and ultimately DVT. Interestingly, the present study showed that pulmonary infection was also an independent predictor of IDDVT. Several possible mechanisms may explain the link between pneumonia and DVT. An animal study in which platelets were extracted from wild-type mice and transplanted into Toll-like receptor (TLR)-4-deficient mice that then received either bacteria-produced lipopolysaccharide (LPS) or saline injections revealed that LPS initiates thrombosis via TLR-4 [41]. Furthermore, TF may contribute to thrombosis in pneumonia patients [42]. For example, TF protein levels in pulmonary edema fluid of acute lung injury patients are 100 times higher than their simultaneous plasma samples [43]. This finding provides another mechanism by which the infected alveolar epithelium may initiate thrombosis via the upregulation of TF. Furthermore, Levi et al. [44] showed that the protein C system is important during the coagulation/inflammatory response and that impairments in the activation of this system may lead to thrombosis in pneumonia patients. Taken together, these findings suggest that the process by which pneumonia causes DVT is complex and likely involve complementary actions of several mechanisms. Further research is required to validate these mechanisms.

Many models are available to predict DVT in stroke patients. The IMPROVE score is a model for predicting the risk of thrombosis in acutely ill hospitalized patients [21], whereas our model was primarily developed for stroke patients in common wards. The Padua score was empirically generated and has been verified in a cohort study [22]. However, relative to the two abovementioned models, our model is so straightforward that it is possible to understand it at a glance while still being being comprehensive. And our prediction object is IDDVT that few studies has predicted so far. The Liu score is a clinical risk assessment for predicting the incidence rate of DVT in patients with acute stroke within 2 weeks, which was developed using a multicenter prospective cohort with a large sample size [2]. Compared to the Liu score, we added two additional factors, pulmonary infection and atrial fibrillation, into a nomogram model developed specifically to predict the risk of early IDDVT in hospitalized patients with stroke. With the currently available prediction tools, nomograms represent a vital component of the modern medical decision-making model due to their high accuracy and good discrimination characteristics [19, 45]. To our knowledge, there are few models to predict early IDDVT. This nomogram is the first developed model for predicting the risk of IDDVT in patients with AIS. Compared to the IMPROVE score (AUROC = 0.750), Liu score (AUROC = 0.760), and Padua score (AUROC = 0.752) for predicting IDDVT in patients with AIS, the present nomogram model exhibited the best predictive accuracy for the occurrence of IDDVT (AUROC = 0.820). Additionally, the nomogram was validated using a larger cohort for IDDVT prediction in AIS patients.

The present study has several limitations that should be noted. First, the final nomogram was established based on a retrospective analysis of a single-center database. Thus, it is necessary to validate the generalizability of the final nomogram in other centers, and a prospective study is needed to further confirm its reliability. Second, the database used in the present study did not include potential predictors previously shown to affect DVT, such as ABO blood type, and sleep apnea [46, 47]; these markers are not routinely tested for in the clinical practice of AIS patients. Third, the effect of different dose levels of anticoagulation (prophylactic doses versus therapeutic doses) was not analyzed in our study. Fourth, the final nomogram was applicable to AIS patients who did not have previous DVT. Finally, the laboratory variables included in the nomogram were not dynamically measured.

## Conclusion

In conclusion, the present study established and validated a reliable nomogram with good accuracy and discrimination for the individualized prediction of the risk of early IDDVT. And this score was applicable to AIS patients who did not have previous DVT. This nomogram is an easy-to-use tool for the early diagnosis and prevention of IDDVT and may be helpful for clinicians when making decisions and implementing effective preventive interventions according to the individual risks of specific patients.

## Data Availability

The data that support the findings of this study are available on request from the corresponding author. Any data intended for sharing will be de-identified.

## Abbreviations

AIS: Acute ischemic stroke
ALB: Albumin
AUROC: Area under the receiver operating characteristic curve
CI: Confidence interval
CRP: C-reactive protein
DVT: Deep venous thrombosis
Hcy: Homocysteine
hs-CRP: High-sensitivity C-reactive protein
IDDVT: Isolated distal deep vein thrombosis
NIHSS: National Institutes of Health Stroke Scale score
OR: Odds ratio
SCr: Serum creatinine concentration
TF: Tissue factor
TIA: Transient ischaemic attacks
VTE: Venous thrombosis

## References

[1] Ogata T, Yasaka M, Wakugawa Y, Inoue T, Ibayashi S, Okada Y (2008). Deep venous thrombosis after acute intracerebral hemorrhage. J Neurol Sci.

[2] Liu LP, Zheng HG, Wang DZ, Wang YL, Hussain M, Sun HX, Wang AX, Zhao XQ, Dong KH, Wang CX (2014). Risk assessment of deep-vein thrombosis after acute stroke: a prospective study using clinical factors. CNS neuroscience & therapeutics.

[3] Collaboration CCiLOsaST (2014). Effect of intermittent pneumatic compression on disability, living circumstances, quality of life, and hospital costs after stroke: secondary analyses from CLOTS 3, a randomised trial. Lancet Neurology.

[4] Kamran SI, Downey D, Ruff RL (1998). Pneumatic sequential compression reduces the risk of deep vein thrombosis in stroke patients. Neurology.

[5] Kelly J, Rudd A, Lewis RR, Coshall C, Moody A, Hunt BJ (2004). Venous thromboembolism after acute ischemic stroke: a prospective study using magnetic resonance direct thrombus imaging. Stroke.

[6] de Freitas GR, Nagayama M (2009). Deep venous thrombosis after intracerebral hemorrhage, gender and ethnicity: a challenge for therapeutic approaches. Cerebrovascular diseases (Basel, Switzerland).

[7] Brandstater ME, Roth EJ, Siebens HC (1992). Venous thromboembolism in stroke: literature review and implications for clinical practice. Archiv Phys Med Rehab.

[8] Turpie AG, Levine MN, Hirsh J, Carter CJ, Jay RM, Powers PJ, Andrew M, Magnani HN, Hull RD, Gent M (1987). Double-blind randomised trial of Org 10172 low-molecular-weight heparinoid in prevention of deep-vein thrombosis in thrombotic stroke. Lancet (London, England).

[9] Palareti G, Schellong S (2012). Isolated distal deep vein thrombosis: what we know and what we are doing. J Thrombosis Haemostasis.

[10] Cogo A, Lensing AW, Prandoni P, Hirsh J (1993). Distribution of thrombosis in patients with symptomatic deep vein thrombosis. Implications for simplifying the diagnostic process with compression ultrasound. Arch Intern Med.

[11] Galanaud JP, Sevestre-Pietri MA, Bosson JL, Laroche JP, Righini M, Brisot D, Boge G, van Kien AK, Gattolliat O, Bettarel-Binon C (2009). Comparative study on risk factors and early outcome of symptomatic distal versus proximal deep vein thrombosis: results from the OPTIMEV study. Thromb Haemost.

[12] Robert-Ebadi H, Righini M (2017). Should we diagnose and treat distal deep vein thrombosis?. Hematol Am Soc Hematol Educ Program.

[13] Galanaud JP, Quenet S, Rivron-Guillot K, Quere I, Sanchez Munoz-Torrero JF, Tolosa C, Monreal M (2009). Comparison of the clinical history of symptomatic isolated distal deep-vein thrombosis vs. proximal deep vein thrombosis in 11 086 patients. J Thrombosis Haemostasis.

[14] Palareti G (2014). How I treat isolated distal deep vein thrombosis (IDDVT). Blood.

[15] Spencer FA, Kroll A, Lessard D, Emery C, Glushchenko AV, Pacifico L, Reed G, Gore JM, Goldberg RJ (2012). Isolated calf deep vein thrombosis in the community setting: the Worcester venous thromboembolism study. J Thromb Thrombolysis.

[16] Wille-Jorgensen P, Jorgensen LN, Crawford M (2005). Asymptomatic postoperative deep vein thrombosis and the development of postthrombotic syndrome. A systematic review and meta-analysis. Thromb Haemost.

[17] Kappelle LJ (2011). Preventing deep vein thrombosis after stroke: strategies and recommendations. Curr Treat Options Neurol.

[18] Cappellari M, Turcato G, Forlivesi S, Zivelonghi C, Bovi P, Bonetti B, Toni D (2018). STARTING-SICH Nomogram to predict symptomatic Intracerebral hemorrhage after intravenous thrombolysis for stroke. Stroke.

[19] Hogue O, Fernandez HH, Floden DP (2018). Predicting early cognitive decline in newly-diagnosed Parkinson's patients: a practical model. Parkinsonism Relat Disord.

[20] Rosenberg D, Eichorn A, Alarcon M, McCullagh L, McGinn T, Spyropoulos AC (2014). External validation of the risk assessment model of the international medical prevention registry on venous thromboembolism (IMPROVE) for medical patients in a tertiary health system. J Am Heart Assoc.

[21] Spyropoulos AC, Anderson FA, FitzGerald G, Decousus H, Pini M, Chong BH, Zotz RB, Bergmann JF, Tapson V, Froehlich JB (2011). Predictive and associative models to identify hospitalized medical patients at risk for VTE. Chest.

[22] Barbar S, Noventa F, Rossetto V, Ferrari A, Brandolin B, Perlati M, De Bon E, Tormene D, Pagnan A, Prandoni P (2010). A risk assessment model for the identification of hospitalized medical patients at risk for venous thromboembolism: the Padua prediction score. J Thrombosis Haemostasis.

[23] Iasonos A, Schrag D, Raj GV, Panageas KS (2008). How to build and interpret a nomogram for cancer prognosis. J Clin Oncol.

[24] Enga KF, Rye-Holmboe I, Hald EM, Lochen ML, Mathiesen EB, Njolstad I, Wilsgaard T, Braekkan SK, Hansen JB (2015). Atrial fibrillation and future risk of venous thromboembolism:the Tromso study. J Thrombosis Haemostasis.

[25] Wang CC, Lin CL, Wang GJ, Chang CT, Sung FC, Kao CH (2015). Atrial fibrillation associated with increased risk of venous thromboembolism. A population-based cohort study. Thromb Haemost.

[26] Prandoni P (2009). Venous and arterial thrombosis: two aspects of the same disease?. Eur J Internal Med.

[27] Khan AA, Lip GYH (2019). The prothrombotic state in atrial fibrillation: pathophysiological and management implications. Cardiovasc Res.

[28] Kawase K, Okazaki S, Toyoda K, Toratani N, Yoshimura S, Kawano H, Nagatsuka K, Matsuo H, Naritomi H, Minematsu K (2009). Sex difference in the prevalence of deep-vein thrombosis in Japanese patients with acute intracerebral hemorrhage. Cerebrovascular diseases (Basel, Switzerland).

[29] White RH, Zhou H, Romano PS (2003). Incidence of symptomatic venous thromboembolism after different elective or urgent surgical procedures. Thromb Haemost.

[30] Silverstein MD, Heit JA, Mohr DN, Petterson TM, O'Fallon WM, Melton LJ (1998). Trends in the incidence of deep vein thrombosis and pulmonary embolism: a 25-year population-based study. Arch Intern Med.

[31] Heit JA (2015). Epidemiology of venous thromboembolism. Nat Rev Cardiol.

[32] Dentali F, Pegoraro S, Barco S, di Minno MN, Mastroiacovo D, Pomero F, Lodigiani C, Bagna F, Sartori M, Barillari G (2016). OC-01 - Clinical history of cancer patients with isolated distal deep vein thrombosis: a multicenter cohort study. Thrombosis Res.

[33] Dentali F, Pegoraro S, Barco S (2017). Clinical course of isolated distal deep vein thrombosis in patients with active cancer: a multicenter cohort study. J Thromb Haemost.

[34] Galanaud JP, Sevestre MA, Pernod G, Genty C, Richelet S, Kahn SR, Boulon C, Terrisse H, Quere I, Bosson JL (2017). Long-term outcomes of cancer-related isolated distal deep vein thrombosis: the OPTIMEV study. J Thrombosis Haemostasis.

[35] Hisada Y, Mackman N (2017). Cancer-associated pathways and biomarkers of venous thrombosis. Blood.

[36] Kucuk O, Kwaan HC, Gunnar W, Vazquez RM (1985). Thromboembolic complications associated with L-asparaginase therapy. Etiologic role of low antithrombin III and plasminogen levels and therapeutic correction by fresh frozen plasma. Cancer.

[37] Knight R, DeLap RJ, Zeldis JB (2006). Lenalidomide and venous thrombosis in multiple myeloma. N Engl J Med.

[38] Barsoum MK, Heit JA, Ashrani AA, Leibson CL, Petterson TM, Bailey KR (2010). Is progestin an independent risk factor for incident venous thromboembolism? A population-based case-control study. Thromb Res.

[39] Dennis M, Sandercock P, Reid J, Graham C, Murray G, Venables G, Rudd A, Bowler G (2011). Can clinical features distinguish between immobile patients with stroke at high and low risk of deep vein thrombosis? Statistical modelling based on the CLOTS trials cohorts. J Neurol Neurosurg Psychiatry.

[40] Hitos K, Cannon M, Cannon S, Garth S, Fletcher JP (2007). Effect of leg exercises on popliteal venous blood flow during prolonged immobility of seated subjects: implications for prevention of travel-related deep vein thrombosis. J Thrombosis Haemostasis.

[41] Stark RJ, Aghakasiri N, Rumbaut RE (2012). Platelet-derived toll-like receptor 4 (Tlr-4) is sufficient to promote microvascular thrombosis in endotoxemia. PLoS One.

[42] Rijneveld AW, Weijer S, Bresser P, Florquin S, Vlasuk GP, Rote WE, Spek CA, Reitsma PH, van der Zee JS, Levi M (2006). Local activation of the tissue factor-factor VIIa pathway in patients with pneumonia and the effect of inhibition of this pathway in murine pneumococcal pneumonia. Crit Care Med.

[43] Bastarache JA, Wang L, Geiser T, Wang Z, Albertine KH, Matthay MA, Ware LB (2007). The alveolar epithelium can initiate the extrinsic coagulation cascade through expression of tissue factor. Thorax.

[44] Levi M, Dorffler-Melly J, Reitsma P, Buller H, Florquin S, van der Poll T, Carmeliet P (2003). Aggravation of endotoxin-induced disseminated intravascular coagulation and cytokine activation in heterozygous protein-C-deficient mice. Blood.

[45] Touijer K, Scardino PT (2009). Nomograms for staging, prognosis, and predicting treatment outcomes. Cancer.

[46] Li D, Pise MN, Overman MJ, Liu C, Tang H, Vadhan-Raj S, Abbruzzese JL (2015). ABO non-O type as a risk factor for thrombosis in patients with pancreatic cancer. Cancer Med.

[47] Lippi G, Mattiuzzi C, Franchini M (2015). Sleep apnea and venous thromboembolism. A systematic review. Thromb Haemost.

